# A Novel and Simplified MSI Approach to Predicting the Long-term Cardiac Function of STEMI

**DOI:** 10.2174/0115734056375695250707070025

**Published:** 2025-07-23

**Authors:** Qifei Xie, Meiling Nie, Feifei Zhang, Xiaoliang Shao, Jianfeng Wang, Juan Song, Yuetao Wang

**Affiliations:** 1 Department of Nuclear Medicine, The Third Affiliated Hospital of Soochow University, Changzhou, Jiangsu Province, China; 2 Department of Cardiology,, Xiamen Cardiovascular Hospital, Xiamen University, Xiamen, China; 3 Institute of Clinical Translation of Nuclear Medicine and Molecular Imaging, Soochow University, Changzhou, Jiangsu Province, China

**Keywords:** STEMI, SPECT, Myocardial salvage index, Cardiac function, Prognosis, PCI, TPD, CAG

## Abstract

**Introduction:**

The Myocardial Salvage Index (MSI) is a valuable indicator in ST-segment Elevation Myocardial Infarction (STEMI) treated with Percutaneous Coronary Intervention (PCI), yet challenges exist in its acquisition. This study aims to calculate MSI using Coronary Angiography (CAG) and myocardial perfusion imaging, and further investigate its correlation with long-term cardiac function.

**Methods:**

In 203 STEMI, the myocardium at risk was measured through CAG using the Bypass Angioplasty Revascularization Investigation Myocardial Jeopardy Index (BARI) score. The infarcted myocardium was measured by the Total Perfusion Deficit (TPD) obtained in Myocardial Perfusion Imaging (MPI) after PCI. MSI was computed as (BARI score–TPD)/BARI score. Long-term cardiac function was assessed via echocardiography.

**Results:**

The MSI is notably associated with the long-term cardiac function [EF: Beta = 16 (13, 20), P < 0.00; LVD: Beta = -7.3 (-9.3, -5.3), P < 0.001]. TIMI flow grades 2-3 demonstrate a superior MSI compared to grades 0-1 [0.78 (0.32) *vs.* 0.61 (0.38), P = 0.002]. TIMI flow grades have an impact on MSI [Beta = 0.08 (0.04, 0.13), P < 0.001]. Compared to patients with a Killip grade of < 2, those with a grade ≥ 2 exhibit a lower MSI [0.69 (0.35) *vs.* 0.48 (0.42), p = 0.005]. The Killip classification has an impact on MSI [Beta = -0.12(-0.19, -0.04), P = 0.003].

**Discussion:**

The study indicates the pivotal role of MSI in predicting long-term cardiac function in STEMI, compares the advantages and limitations of SPECT, CMR, and hybrid SPECT/CAG methods, analyzes the impact of residual blood flow and acute heart failure on MSI, and highlights current technological challenges and future research directions.

**Conclusion:**

CAG combining MPI after PCI can be used to obtain MSI. MSI is linked to long-term cardiac function. The amount of antegrade flow before PCI and the initial cardiac function upon admission significantly influence MSI.

## 1 INTRODUCTION

Coronary Artery Disease (CAD) is highly prevalent in China, imposing a significant burden on public health and healthcare expenditures [1]. Acute Myocardial Infarction (MI), a severe manifestation of CAD, requires timely restoration of blood flow to salvage myocardium and prevent further damage. The Myocardial Salvage Index (MSI) a metric that quantifies the proportion of salvaged myocardium relative to the myocardium at risk has emerged as a valuable tool for assessing treatment efficacy and predicting long-term prognosis [2-4].

The gold standard method for obtaining MSI is through the use of two Single-Photon Emission Computed Tomography (SPECT) scans. The first SPECT examination requires the injection of a radioactive substance before blood flow is restored in the patient, followed by a scan within the effective period of the medication to obtain the Area At Risk (AAR), which denotes the myocardial territory subtended by the occluded coronary artery during acute ischemia. It represents the maximal extent of potentially salvageable tissue if timely reperfusion is achieved, and serves as the denominator in Myocardial Salvage Index (MSI) calculations. The second SPECT examination then requires another routine scan after the patient has received treatment and their condition has stabilized to determine the quantity of infarcted myocardium [2-4]. This approach is cumbersome and often impractical in clinical settings due to the need for pre-reperfusion radiopharmaceutical injection and prolonged symptom-to-balloon time [4, 5].

This study proposes a novel method combining Coronary Angiography (CAG) with MPI to assess MSI. By leveraging CAG to estimate the myocardial at-risk tissue and MPI to quantify infarct size, this study aims to provide a practical and reliable alternative for evaluating myocardial salvage. It further investigates the correlation between MSI and long-term cardiac function, as well as the major factors influencing MSI. This approach has the potential to enhance clinical decision-making and improve patient outcomes in the management of acute MI.

## 2 METHODS

### 2.1 Population

Utilizing data from the Xiamen Cardiovascular Hospital Information Registry Database, this research was a retrospective analysis spanning the period from August 2019 to August 2023.

As shown in the study flowchart, the diagnostic criteria for myocardial infarction are consistent with the Fourth Universal Definition of Myocardial Infarction. The patients underwent routine MPI following Percutaneous Coronary Intervention (PCI) and had over 6 months' worth of Ultrasonic Cardiogram (UCG) results after PCI. In total, 529 patients were included in the analysis. After undergoing artificial secondary screening, a total of 367 individuals were accounted for. The study excluded the following patients from participation: (i) patients with a history of myocardial infarction if they were mistakenly classified based on the combined electrocardiogram findings, (ii) patients with a history of PCI or CABG, (iii) patients who received pharmacological thrombolysis instead of direct PCI, (iv) patients undergoing treatment that includes CABG, (v) patients who experienced a recurrent myocardial infarction prior to MPI, and (vi) patients with unavailable or indeterminate results of coronary angiography for determining the culprit lesion. The specific number of people and methods for selection can be found in Fig. (**1**).

### 2.2 Coronary Angiography

All patients were administered aspirin and P2Y_12_ receptor antagonists as a loading dose and received anticoagulant medications, including unfractionated heparin, low-molecular-weight heparin, or rivaroxaban prior to the surgical procedure.

The PCI treatment options consisted of simple balloon angioplasty, drug-coated balloon angioplasty, and drug-eluting stent implantation. Each patient exhibited a definite culprit lesion, and post-PCI residual stenosis was less than 30%.

The residual blood flow, including antegrade flow and collateral flow, before PCI treatment was assessed. The TIMI grading system was employed to assess antegrade flow of the culprit vessel [6]. Collateral flow was assessed via Rentrop classification (grade 0 indicating the absence of visually identifiable collateral vessels to grade 3 indicating complete retrograde filling of the IRA to the site of occlusion) [7]. Collateral flow was categorized into three groups: the no collateral group, the small collateral group (grade 1), and the large collateral group (grade 2-3).

Two board-certified interventional cardiologists (each with >10 years of experience in coronary angiography analysis), who were blinded to patients' clinical outcomes and other imaging results (including SPECT), independently analyzed and scored the coronary angiography findings using the BARI scoring system. Discrepancies (a difference of >10% in the scores) were resolved by consensus with a third senior cardiologist.

### 2.3 Myocardial Perfusion Imaging

The D-SPECT (Spectrum Dynamics Medical Ltd) was utilized for MPI. A dose of 0.1 mCi (3.7 MBq)/kg of the imaging agent, 99mTc-sestamibi, was administered to all patients during the stable period after PCI (mean duration: 6.6 ± 8.9 months). Scanning was conducted 1 hour after drug injection. Image reconstruction was performed using the SD method [8], with the OMES method used if reconstruction failed.

Image analysis was performed using Quantitative Perfusion SPECT-Quantitative SPECT (QPS Version 2017, Cedars-Sinai Medical Center). Physicians manually adjusted contours when automated detection failed (e.g., apical underestimation or extracardiac inclusion). TPD was calculated automatically through software using a 17-segment model, which represents the integrated extent and severity of the defects, to quantify infarction.

### 2.4 Myocardial Salvage Index Calculation

In all patients, contrast agents were reinjected after blood flow was restored after PCI to view the coronary artery. Visual assessment of this CAG was obtained immediately after PCI completion. The Bypass Angioplasty Revascularization Investigation Myocardial Jeopardy Index (BARI) score was used to calculate the amount of myocardium at risk. As shown in Fig. (**2**), an examination was conducted to determine the sizes of the distal and main branches of the Left Anterior Descending artery (LAD), Left Circumflex artery (LCX), and Right Coronary artery (RCA). The main branches consist of the diagonal branch, the anterior interventricular branch, the obtuse marginal branch, the posterior descending branch, and the left circumflex branch. Scoring was performed based on the thickness and length of each. For instance, if the length of the left ventricle were divided into three segments from the basal segment to the apex, a score of 1 point would be given if less than one-third is reached, and 3 points would be given if more than two-thirds is reached. Thickness and branching patterns were evaluated in relation to the conditions of the three vessels. If there were clearly visible collateral blood vessels, a deduction of 1 point should be made from the scoring method mentioned above, with a maximum score limit of 2 points.

All scores that are solely influenced by the culprit lesion were divided by the global score. The final amount ratio was myocardium at risk to total left ventricular myocardium. This ratio, when multiplied by 100% was the AAR.

The myocardial salvage area was calculated by subtracting the infarct size (derived from TPD) from the Area At Risk (AAR, quantified by BARI score). The Myocardial Salvage Index (MSI) was then computed as (eq. **1**) (as shown in Fig. (**2**):

**Table:** 

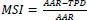	(1)

where:

• ​**AAR** = Area at risk (The percentage of already infarcted myocardium relative to the total left ventricular myocardium)

• ​**TPD** = Total Perfusion Deficit (The percentage of myocardium at risk of infarction [including already infarcted tissue] relative to the total left ventricular myocardium)

• ​**Salvage area** = AAR - TPD (The percentage of salvaged myocardium

relative to the total left ventricular myocardium)

### 2.5 Cardiac Functional Indicators and Outcome Events

The cardiac functional indicators of patients were collected at least six months after PCI from an Ultrasonic Cardiogram (UCG). The main indicators to be collected were left ventricular end-diastolic size (LVD) and left ventricular ejection fraction (EF).

Major Adverse Cardiac Events (MACE) encompass repeated PCI in the same culprit vessel, progression of stenosis in other vessels exceeding 10%, and total stenosis reaching 75% or more. Additionally, MACE includes recurrent myocardial infarction and all-cause death. ‘Hard Events’ are defined as recurrent myocardial infarction and all-cause death only.

### 2.6 Statistical Analysis

All continuous indicators are presented as the mean±standard deviation, while discrete indicators are reported as n (%). Continuous variables were assessed using the ​independent t-test or ​Mann-Whitney U test, depending on the adherence to a normal distribution and equal variance, with consideration given to the sample size. The Pearson chi-square test or Fisher's exact test was employed, depending on the sample size and expected frequencies in each cell of the contingency table for categorical variables. To establish multivariable models and assess the impact of significant indicators on the salvage index, multiple linear regression models were utilized. The ROC curve was used to determine the optimal threshold and diagnostic performance of the salvage index for long-term postoperative cardiac function in patients.

All analyses were performed using the R package (version 4.3.1).

### 2.7 Ethical Approval and Consent

All procedures were conducted in accordance with the ethical standards of the Institutional Research Committee and with the 1964 Declaration of Helsinki and its later amendments or comparable ethical standards. The study protocol was approved by the Ethics Committee of Xiamen Cardiovascular Hospital (Ethical No.2020YLK14).

## 3 RESULT

### 3.1 Baselines

#### 3.1.1 Difference Between STEMI and NSTEMI

In comparison to Non-ST-Elevation Myocardial Infarction (NSTEMI), the TPD is generally greater in STEMI. Conversely, the MSI is lower. Both indices showed greater variability in STEMI. Moreover, the long-term UCG revealed worse heart function, and more ‘Hard Events’ (five cases of recurrent myocardial infarction and three deaths in STEMI, one recurrent myocardial infarction in NSTEMI).

Additionally, NSTEMI patients exhibited a significantly higher number of multi-vessel disease and complex lesions compared to STEMI, leading subsequent studies to predominantly focus on STEMI. The specific baseline data can be found in (Table **1**).

#### 3.1.2 Interobserver Agreement for BARI Score

Patients with normal MPI and those with TPD exceeding 50% were excluded from the calculation of the BARI score. The MSI scores of these patients were calculated directly as either 0 or 1. A total of 134 patients' BARI scores were calculated. To ensure the reliability and repeatability of the computed results, the BARI score was calculated by two experienced cardiologists who have been involved in coronary PCI treatment for many years. The comparison between the two evaluations is presented in Fig. (**3**).

### 3.2 Correlation of MSI with Long-term Cardiac Function

#### 3.2.1 Effect of MSI on Long-term Cardiac Function

**Fig. (4):**
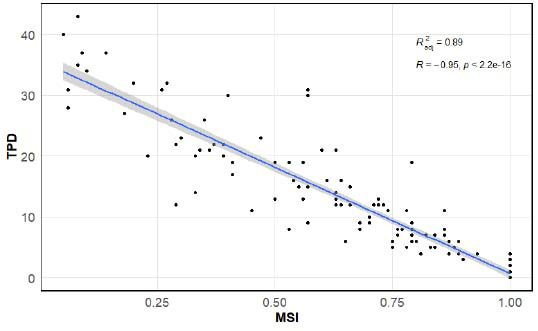
In patients with MSI > 0, MSI and TPD exhibit a significant correlation, as assessed by the Spearman correlation coefficient. Radj2​ serves as the coefficient of determination in linear regression models.

The UCGs in this study were obtained more than six months (Mean ± SD: 10.3 ± 4.2) after PCI, including LVD and EF. Separate regression models were constructed for TPD and MSI to account for their high correlation (Fig. **4**), as determined by multi-factor analysis, thereby mitigating issues of multicollinearity. The outcome variable is the index of Long-term UCG. Age is considered a continuous variable in both models. Symptom-to-balloon time group, TIMI grade, Collateral Flow Group, Multi-vessel, and Killip grade were categorized as ordinal variables. Multi-vessel represents the presence of multiple coronary artery lesions. In the absence of severe stenosis in the coronary arteries other than the culprit vessel, it is scored as 0. It can have a maximum of 2, resulting in three levels in total. The results are shown in (Tables **2** and **3**).

#### 3.2.2 Effectiveness of MSI in Predicting Cardiac Function

For ease of calculation, the normal values of LVD for both genders were set below 55mm, while the normal values of EF were set at or above 50%. Based on this, it can be observed that MSI provides a certain level of accuracy in estimating the long-term cardiac function of patients, and its predictive ability for EF values is superior to that of LVD. The optimum threshold for determining whether EF will decrease is identified as 0.585. At this threshold, specificity is high, and the sensitivity is relatively good. Similarly, the optimal threshold for determining whether LVD will exceed the normal value was found to be 0.644. At this threshold, the specificity remains acceptable, but the sensitivity is relatively low. Fig. (**5**) provides detailed information on these findings.

### 3.3 Major Factors Affecting MSI

#### 3.3.1 Antegrade Flow or Collateral Flow Before PCI

The residual blood flow has a significant impact on the size of the infarction. It is evident that patients with larger antegrade flow experience a significant increase in both the number of myocardial infarctions and salvaged myocardium in the final stage. Additionally, their cardiac function improves after six months, and the size of the ventricle is relatively smaller. While collateral vessels are also recognized as a factor affecting myocardial infarction, their formation is rarely observed in cases of STEMI. Due to limitations in sample size, this study conducted a subgroup analysis and provided baseline conditions for reference. The specific results are presented in Table **4**.

#### 3.3.2 Symptom-to-Balloon Time

The duration from the onset of chest pain to the restoration of blood flow is a recognized influential factor in myocardial infarction. Interestingly, in cases where the patient's symptoms are typical and persistent, there is a delay in the restoration of blood flow, leading to a gradual decline in the number of infarcted myocardial cells, myocardial salvage, and long-term cardiac function. However, in cases where the onset of chest pain is further delayed, a significant proportion of patients experience improvement in their symptoms. Nevertheless, if not promptly treated, chest pain may recur in this group of patients, leading to intermittent episodes. Although the exact cause of improvement in these cases of chest pain remains unknown, it is hypothesized that the mechanism of ischemic preconditioning may play a vital role. The specific results are presented in Table **5**.

#### 3.3.3 Acute Heart Failure Condition Before PCI

The specific values of the patient's heart function prior to the onset of the disease could not be determined. Furthermore, the measurement of the patient's heart function in the days or even hours after the surgery varies. However, it is imperative to consider the patient's baseline heart function in relation to their long-term heart function. For individuals with STEMI, the Killip classification offers a semi-quantitative approximation of the patient's heart function upon admission, unlike in cases of chronic heart failure. It is evident that the heart function upon admission not only impacts the patient's long-term heart function but also influences the occurrence of myocardial infarctions and the salvage index. The specific results are shown in Table **6**.

#### 3.3.4 Multi-Factor Regression Analysis

After incorporating the mentioned confounding factors, such as age, gender, hypertension, diabetes, and hyperlipidemia, and conducting a multiple linear regression analysis with the outcome variable as MSI, it was discovered that in addition to the established factors concerning blood flow, the existence of acute heart failure upon admission also significantly affects the efficacy of myocardial salvage. The specific results are presented in Table **7**.

## 4 DISCUSSION

### 4.1 Key Findings and Their Significance

This study investigated the role of the MSI in predicting long-term cardiac function in patients with ​STEMI. The results demonstrate that MSI has a significant and strong impact on long-term cardiac function, surpassing the predictive value of other risk factors analyzed in this study.

MSI quantifies the proportion of salvaged myocardium relative to the ​Area At Risk (AAR) following reperfusion therapy [3, 9, 10]. Although MSI has been validated in numerous studies, its widespread clinical application has been limited by challenges in accurately assessing the AAR. This study adopted a novel approach by integrating ​ SPECT and ​CAG to calculate MSI, demonstrating its correlation with long-term cardiac function. This combination of functional and anatomical imaging provides a more comprehensive and clinically relevant assessment of myocardial salvage.

### 4.2 SPECT *vs.* CMR *vs.* Hybrid SPECT/CAG: A Systematic Clinical Evaluation

The gold standard for MSI calculation is based on SPECT-derived ​myocardial perfusion imaging (MPI) [4, 11, 12]. However, SPECT has limitations in assessing the AAR, which is critical for MSI calculation. This requires the injection of radiopharmaceuticals prior to reperfusion and immediate imaging post-reperfusion to determine the AAR, followed by a second MPI session after the patient stabilizes to assess the infarct size. This process is not only cumbersome but may also delay timely myocardial salvage, making it inconsistent with clinical workflows.

In contrast, ​Cardiac Magnetic Resonance (CMR) imaging offers high spatial resolution and the ability to retrospectively quantify the AAR using myocardial edema as a surrogate without radiation exposure [4, 13, 14]. CMR also enables the precise quantification of infarct size through ​delayed enhancement imaging, making it a robust tool for MSI calculation [5, 15]. With the recent availability of advanced techniques, such as ​native T1 mapping, ​T2 mapping [16], T2* mapping [17-19], and ​post-contrast T1 mapping (used to derive Extracellular Volume fraction [ECV] mapping) [20], CMR provides deeper insights into pathophysiological processes, such as the evolution of myocardial edema in the first week after STEMI.

In the first week following STEMI, the evolution of myocardial edema undergoes several stages: during the acute ischemic phase (0-6 hours), intracellular edema begins to form; during the reperfusion phase (6-24 hours), edema rapidly intensifies; during the acute inflammatory phase (24-72 hours), edema peaks; during the repair phase (3-7 days), edema gradually subsides; and during the scar formation phase (after 1 week), edema largely resolves [19, 21]. Techniques such as T2-weighted CMR, T1 mapping, and ECV mapping can dynamically monitor this evolution, providing valuable insights into the processes of myocardial injury and repair.

Despite these advantages, the widespread clinical adoption of CMR is limited by several factors.​ CMR is expensive and requires specialized equipment and expertise, limiting its availability in many healthcare settings. CMR requires patients to remain still and perform breath-holds during the scan, which can be challenging for STEMI patients, especially those with heart failure or respiratory distress. Additionally, imaging patients with arrhythmias or elevated heart rates can be difficult. CMR scans typically take 30-60 minutes, which may pose logistical challenges in busy clinical environments.​ The dynamic evolution of myocardial edema and the detection of infarct size over time pose a significant challenge for AAR estimation and MSI calculation. Both the AAR and infarct size can vary depending on the timing of imaging, making it difficult to determine the optimal time window for MSI assessment. This temporal variability raises questions about whether a single time point is sufficient for accurate MSI calculation or if multiple assessments are needed to capture the dynamic changes in myocardial injury and salvage. Further research is required to establish standardized protocols for timing MSI measurements in clinical practice.

In contrast, our SPECT-CAG method offers several advantages. SPECT is more widely available and less expensive, making it a more practical option for routine clinical use.​ Additionally, it does not require the same level of technical expertise or specialized equipment as CMR. This method can be integrated into existing workflows without the need for additional imaging sessions, thereby reducing the burden on patients and healthcare providers.

Additionally, our method of estimating infarct size using SPECT-based MPI is influenced by post-infarction pathophysiological changes. The true infarct size can only be determined after the patient’s pathophysiological state stabilizes (e.g., after 1 week). In contrast, AAR estimation using CAG is not restricted by a time window and can be performed at any time. However, SPECT also has notable limitations. Its lower spatial resolution (~4 mm) may affect the accuracy of assessing non-transmural infarctions. Compared to CMR, SPECT exposes patients to higher levels of ionizing radiation.

### 4.3 Factors Influencing MSI

#### 4.3.1 Role of Residual Blood Flow

Residual blood flow to the myocardium supplied by the culprit vessel before PCI is a critical determinant of MSI [22, 23]. Residual blood flow consists of ​antegrade flow and ​collateral flow, each exerting distinct effects on myocardial salvage. Antegrade flow aligns with physiological hemodynamic characteristics, primarily affecting subendocardial perfusion, while collateral flow influences the transmural progression of myocardial infarction from the endocardium to the epicardium [5, 24]. However, the lower spatial resolution of SPECT (~4 mm) and its significantly lower photon flux compared to CT imaging pose challenges in differentiating the morphology of transmural infarction. Therefore, this study was unable to validate the above conclusions. As a result, MSI values obtained via SPECT may show slight variations compared to those obtained using other imaging methods, but the overall trend among different patients should remain consistent.

#### 4.3.2 Role of Acute Heart Failure and Killip Classification

The presence of ​acute heart failure (assessed using the ​Killip classification) upon admission significantly influences MSI and long-term cardiac function [25-30]. Patients with severe acute heart failure typically exhibit smaller MSI, indicating a higher likelihood of myocardial infarction rather than ​myocardial stunning. Myocardial stunning is a reversible condition characterized by transient dysfunction despite normal blood flow, typically resolving within one week after reperfusion [31-33]. However, prolonged ischemia may lead to irreversible myocardial infarction, resulting in worse long-term outcomes. Thus, the Killip classification is an important confounding factor in this study, emphasizing the need to consider baseline cardiac function when interpreting MSI.

### 4.4 Limitations and Future Directions

This study has several limitations inherent to its retrospective design. First, the quality and variability of data documented in medical records, along with potential recall bias among patients, may affect the accuracy of certain variables, such as symptom-to-balloon time. Second, the study did not account for ​complete revascularization, which has been shown to benefit STEMI patients compared to culprit vessel-only revascularization. The variability in reconstruction methods and time intervals among patients who underwent complete revascularization further complicates the analysis of the data.

Additionally, the estimation of AAR using coronary angiography is subjective and may be inaccurate in cases of difficult-to-identify coronary lesions or vascular anatomical anomalies. For example, besides the BARI score used in this study, the ​APPROACH score is another alternative method based on coronary angiography [34], offering greater convenience and reproducibility. However, it also suffers from subjectivity. Selecting different methods to estimate AAR in various scenarios may yield better results.

## CONCLUSION

The MSI can be reliably quantified through the combined use of CAG and post-PCI MPI. This integrated approach provides a clinically practical and cost-effective solution for evaluating myocardial salvage, making it especially suitable for routine clinical practice. MSI quantified by this method shows a strong positive correlation with long-term cardiac functional outcomes. MSI is significantly influenced by two key pretreatment factors: antegrade flow before PCI and initial cardiac function at admission.

## AUTHORS’ CONTRIBUTIONS

The authors confirm their contribution to the paper as follows: W.-Y.T. and S.J. designed the study and obtained the funding support. X.-Q.F. and N.-M.L. analyzed the data and drafted the manuscript. Z.-F.F, S.-X.L., and W.-J.F. assisted in the study design and performed a series of data analyses. All authors have read and approved the article.

## Figures and Tables

**Fig. (1):**
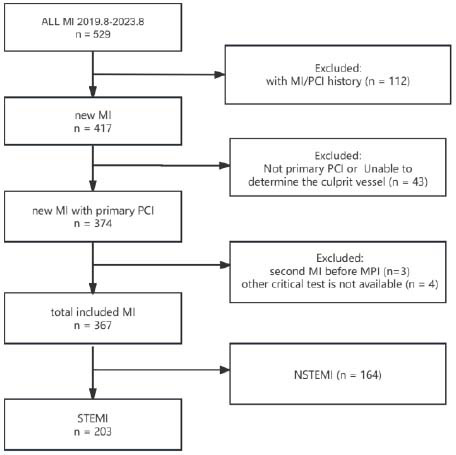
Study flowchart.

**Fig. (2):**
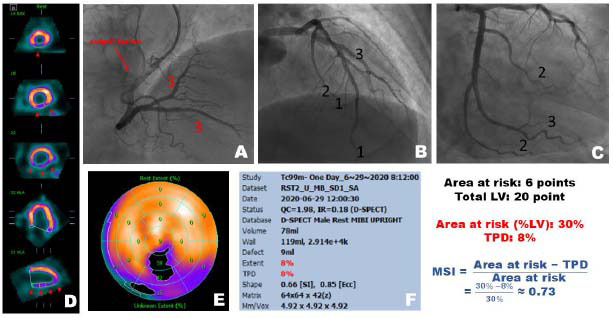
One example combining Coronary Angiography (**A**-**C**) and SPECT (**D-F**) for Myocardial Salvage Index Calculation. The red triangle on the left side of the MPI indicates that the patient’s MI is located in the posterior-inferior wall (**D**), and the coronary angiography on the right side suggests that the culprit lesion (indicated by a red arrow in **A**) is located in the proximal RCA 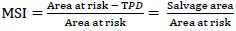.

**Fig. (3):**
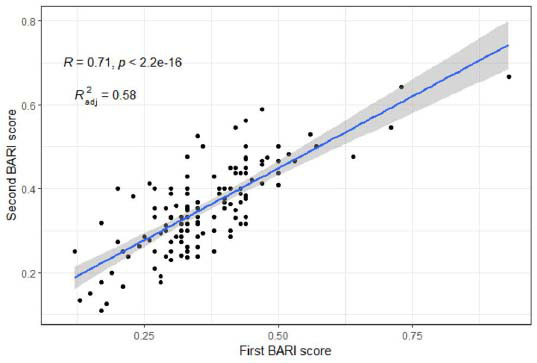
A total of 134 BARI scores of patients were independently calculated by two separate observers. The two observers are unaware of each other’s calculated results, as well as any other medical information about the patients, except for the results of coronary angiography. The evaluation of consistency between the scores calculated by the two observers is depicted in the upper left corner of this figure, denoted as R (Spearman correlation coefficient). R2adj serves as the coefficient of determination in linear regression models.

**Fig. (5):**
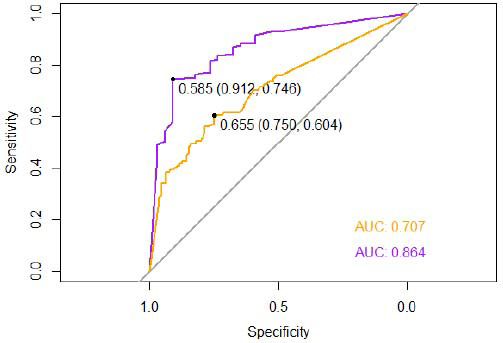
The effectiveness of MSI to evaluate the subsequent cardiac function. An EF value ≥ 50% is considered normal. LVD ≤ 55mm is considered normal. The purple line and AUC value correspond to EF, while the orange line and AUC value correspond to LVD.

**Table 1:** Baseline characteristics, long-term cardiac function, and endpoint event outcomes in STEMI (and NSTEMI) patients.

	**NSTEMI, N = 164**	**STEMI, N = 203**	**p-value^1^**
**Gender**			0.3
Female	26 (16%)	25 (12%)	
Male	138 (84%)	178 (88%)	
Age	63 (11)	60 (13)	0.021
Diabetes	62 (38%)	58 (29%)	0.061
Hypertension	94 (57%)	86 (42%)	0.004
Hyperlipidemia	72 (44%)	106 (52%)	0.11
**Myocardial injury and salvage indices^2^**			
TPD	2.8 (4.4)	13.9 (16.0)	<0.001
MSI	0.91 (0.18)	0.65 (0.37)	<0.001
**Long-term UCG indices^3^**			
LVD (mm)	48 (5)	50 (6)	<0.001
EF (%)	64 (8)	58 (10)	<0.001
**Main Endpoint Event**			
MACE	31 (19%)	49 (24%)	0.2
Hard Event	1 (0.6%)	8 (3.9%)	0.046

All continuous indicators are presented as Mean (SD); Discrete indicators are reported as n (%). ^1^Pearson's Chi-squared test; Wilcoxon rank sum test; Fisher's exact test. ^2^MPI was obtained after PCI (6.9 ± 8.5 months) before the Main endpoint event. ^3^UCG was obtained after PCI (10.3 ± 4.2 months) before the Main endpoint event.

**Table 2:** Multi-variate predictors for long-term cardiac function with TPD.

-	**Long-term EF**			**Long-term LVD**		
	**Beta**	**95% CI** ^1^	**p-value**	**Beta**	**95% CI** ^1^	p-value*
**Age**	0.01	-0.07, 0.10	0.8	-0.05	-0.11, 0.01	0.10
**Gender**	3.0	-0.20, 6.1	0.068	-2.7	-5.0, -0.48	0.019
**Diabetes**	1.0	-1.2, 3.3	0.4	-1.2	-2.9, 0.38	0.13
**Hypertension**	0.40	-1.7, 2.5	0.7	1.8	0.31, 3.4	0.019
**Hyperlipidemia**	1.7	-0.33, 3.7	0.10	-0.71	-2.2, 0.74	0.3
**Symptom-to-balloon time group^2^**	-0.25	-1.1, 0.60	0.6	0.18	-0.43, 0.79	0.6
**TIMI grade**	-0.06	-1.0, 0.88	>0.9	-0.47	-1.1, 0.21	0.2
**Collateral Flow Group**	-0.17	-2.6, 2.2	0.9	-1.1	-2.8, 0.59	0.2
**Multivessel**	-0.76	-2.1, 0.62	0.3	-0.55	-1.5, 0.43	0.3
**Killip grade**	-1.7	-3.3, -0.12	0.036	0.16	-0.97, 1.3	0.8
**TPD**	-0.46	-0.52, -0.39	<0.001	0.18	0.14, 0.23	<0.001

*Using multiple linear regression for modeling. ^1^CI = Confidence Interval. ^2^The time was divided into four groups, as shown in Table **4**.

**Table 3:** Multi-variate predictors for long-term cardiac function with MSI.

-	**Long-term EF**			**Long-term LVD**		
	**Beta**	**95% CI** ^1^	**p-value**	**Beta**	**95% CI** ^1^	**p-value**
**Age**	0.01	-0.08, 0.11	0.8	-0.05	-0.11, 0.01	0.10
**Gender**	1.6	-1.9, 5.1	0.4	-2.2	-4.5, 0.13	0.067
**Diabetes**	1.6	-0.95, 4.1	0.2	-1.5	-3.1, 0.15	0.077
**Hypertension**	0.97	-1.4, 3.3	0.4	1.6	0.10, 3.2	0.039
**Hyperlipidemia**	2.1	-0.18, 4.3	0.072	-0.81	-2.3, 0.65	0.3
**Symptom-to-balloon time group^2^**	-0.47	-1.4, 0.48	0.3	0.25	-0.36, 0.86	0.4
**TIMI grade**	0.29	-0.75, 1.3	0.6	-0.55	-1.2, 0.13	0.11
**Collateral Flow Group**	0.31	-2.4, 3.0	0.8	-1.2	-3.0, 0.50	0.2
**Multivessel**	-0.15	-1.7, 1.4	0.8	-0.77	-1.8, 0.23	0.13
**Killip grade**	-2.3	-4.1, -0.58	0.010	0.33	-0.81, 1.5	0.6
**MSI**	16	13, 20	<0.001	-7.3	-9.3, -5.3	<0.001

*Using multiple linear regression for modeling. ^1^CI = Confidence Interval. ^2^The time was divided into four groups, as shown in Table **4** .

**Table 4:** Coronary angiography findings, msi, and follow-up results in STEMI according to TIMI Grade Before PCI.

-	**Antegrade Flow**		
	**TIMI 0 to 1, N = 148**	**TIMI 2 to 3, N = 55**	**p-value**
**Coronary Angiography Findings**			
Collateral Flow			0.053
Good Collateral Vessels	8 (5.4%)	0 (0%)	
Poor Collateral Vessels	7 (4.7%)	0 (0%)	
No Collateral Vessel	133 (90%)	55 (100%)	
Culprit Vessel			0.2
LAD	74 (50%)	35 (64%)	
LCX	15 (10%)	5 (9.1%)	
LM	1 (0.7%)	1 (1.8%)	
RCA	58 (39%)	14 (25%)	
Multivessel Disease	60 (41%)	21 (38%)	0.8
**Myocardial injury and salvage indices^1^**			
TPD	16.0 (16.9)	8.2 (12.0)	0.002
MSI	0.61 (0.38)	0.78 (0.32)	0.002
**Long-term UCG indices^2^**			
LVD (mm)	51.2 (5.8)	48.3 (6.0)	0.002
EF (%)	57 (11)	61 (9)	0.024
**Main endpoint event**			
MACE	35 (24%)	13 (24%)	>0.9
Hard Event	6 (4.1%)	1 (1.8%)	0.7

*The statistical description and methods for each variable are the same as those in Table **1**. ^1^MPI obtained after PCI (6.6 ± 8.9 months) before the Main endpoint event. ^2^UCG was obtained after PCI (10.4 ± 4.2 months) before the Main endpoint event.

**Table 5:** Effects of symptom time on MSI and follow-up results in STEMI.

-	**Symptom-to-balloon Time^1^**			
	**< 2H, N = 40**	**2-4H, N = 35**	**4-6H, N = 26**	**> 6H, N = 102^2^**
**Myocardial injury and salvage indices**				
TPD	10.1 (11.6)	12.5 (15.7)	17.7 (17.8)	14.9 (17.0)
MSI	0.72 (0.30)	0.69 (0.34)	0.55 (0.43)	0.64 (0.39)
**Long-term UCG indices**				
LVD (cm)	50.1 (5.1)	49.9 (6.0)	50.8 (5.9)	50.6 (6.4)
EF (%)	61 (9)	60 (9)	56 (12)	58 (11)
**Main endpoint event**				
MACE	11 (28%)	7 (20%)	7 (27%)	23 (23%)
Hard Event	2 (5.0%)	3 (8.6%)	0 (0%)	2 (2.0%)

The statistical description and methods for each variable are the same as those in Table **1**. ^1^Continuous chest pain lasting for more than 30 minutes is defined as a valid symptom attack. The time from the initial onset of a valid symptom attack to the time of performing PCI balloon dilation on the blood vessels is defined as Symptom-to-balloon time. ^2^Patients who experience symptoms for more than 4 hours include those with multiple valid symptom episodes, patients with atypical chest pain symptoms, and patients who are unable to determine the time of the first valid symptom or have experienced chest pain multiple times but cannot confirm the existence of valid symptoms.

**Table 6:** Differences in msi and follow-up results based on Killip Grade before PCI.

-	**Assessment of Acute Heart Failure^1^**		
	**Killip Grade < 2, N = 164**	**Killip Grade ≥ 2, N = 39**	**p-value**
**Myocardial injury and salvage indices**			
TPD	11.7 (14.0)	22.9 (20.5)	0.001
MSI	0.69 (0.35)	0.48 (0.42)	0.005
**Long-term UCG indices**			
LVD (mm)	50.0 (5.7)	52.0 (6.9)	0.10
EF (%)	60 (9)	54 (13)	0.005
**Main endpoint event**			
MACE	37 (23%)	11 (28%)	0.5
Hard Event	5 (3.0%)	2 (5.1%)	0.6

The statistical description and methods for each variable are the same as those in Table **1**.

**Table 7:** Multi-variate predictors of MSI.

-	**Beta**	**95% CI^1^**	p-value*
**Gender**	0.05	-0.11, 0.21	0.5
**Age**	0.00	0.00, 0.00	>0.9
**Diabetes**	-0.03	-0.14, 0.08	0.6
**Hypertension**	0.06	-0.04, 0.17	0.2
**Hyperlipidemia**	0.07	-0.03, 0.17	0.2
**Symptom-to-balloon time^2^**	-0.03	-0.07, 0.01	0.2
**Killip grade^3^**	-0.12	-0.19, -0.04	0.003
**TIMI grade^4^**	0.08	0.04, 0.13	<0.001
**Collateral Flow^5^**	0.14	0.03, 0.26	0.017

*Using multiple linear regression for modeling. ^1^CI = Confidence Interval. ^2^The time was divided into four groups, as shown in Table **3**. ^3^According to the Killip classification in the GRACE score, acute heart failure is categorized into four ordered levels based on the condition of lung rales before PCI. ^4^According to the Antegrade Flow velocity during pre-coronary angiography of PCI, it is classified into four levels. ^5^Divided into three ordered groups, as shown in Table **2**.

## Data Availability

The data and supportive information are available within the article.

## LIST OF ABBREVIATIONS

AAR: Area at Risk
APPROCH: A Prospective Pilot Study of Early Cardiac Rehabilitation for Patients with Acute Myocardial Infarction
BARI: Bypass Angioplasty Revascularization Investigation Myocardial Jeopardy Index
CABG: Coronary Artery Bypass Grafting
CAG: Coronary Angiography
CMR: Cardiac Magnetic Resonance Imaging
EF: Left Ventricular Ejection Fraction
LAD: Left Anterior Descending Artery
LCX: Left Circumflex Artery
LVD: Left Ventricular End-Diastolic Size
MACE: Major Adverse Cardiac Events
MSI: Myocardial Salvage Index
PCI: Percutaneous Coronary Intervention
RCA: Right Coronary Artery
SPECT: Single-Photon Emission Computed Tomography
STEMI: ST-Segment Elevation Myocardial Infarction
TIMI: Thrombolysis In Myocardial Infarction
TPD: Total Perfusion Deficit
UCG: Ultrasonic Cardiogram

## STANDARDS OF REPORTING

STROBE guidelines were followed.
